# Immunochemical Characterization of *Acacia* Pollen Allergens and Evaluation of Cross-Reactivity Pattern with the Common Allergenic Pollens

**DOI:** 10.1155/2014/409056

**Published:** 2014-05-18

**Authors:** Mohammad-Hosein Shamsbiranvand, Ali Khodadadi, Mohammad-Ali Assarehzadegan, Seyed Hamid Borsi, Akram Amini

**Affiliations:** ^1^Cancer Research Center of Ahvaz Jundishapur University of Medical Sciences, Ahvaz, Iran; ^2^Department of Immunology, Faculty of Medicine, Ahvaz Jundishapur University of Medical Sciences, Ahvaz 6135715794, Iran; ^3^Department of Internal Medicine, Faculty of Medicine, Ahvaz Jundishapur University of Medical Sciences, Ahvaz, Iran

## Abstract

Pollen from the *Acacia* has been reported as an important source of pollinosis in tropical and subtropical regions of the world. The aim of this study was to characterize the IgE binding protein of *Acacia farnesiana* pollen extract and evaluate cross-reactivity with the most allergenic pollens. In this study, pollen extract was fractionated by SDS-PAGE and the allergenic profile was determined by IgE-immunoblotting and specific ELISA using forty-two *Acacia* allergic patients. Potential cross-reactivity among *Acacia* and selected allergenic plants was evaluated with ELISA and immunoblotting inhibition experiments. There were several resolved protein fractions on SDS-PAGE which ranged from 12 to 85 kDa. Several allergenic protein bands with molecular weights approximately between 12 and 85 kDa were recognized by IgE-specific antibodies from *Acacia* allergic patients in the immunoblot assay. The inhibition by the *Prosopis juliflora* pollen extract was more than those by other pollen extracts. Moreover, the wheal diameters generated by the *Acacia* pollen extract were highly correlated with those of *P. juliflora* pollen extracts. The findings suggest that several proteins such as 15, 23, 45, and 50 kDa proteins could be used as diagnostic and therapeutic reagents for patients allergic to *A. farnesiana* and *P. juliflora*.

## 1. Introduction

*Acacia farnesiana* (*Vachellia farnesiana*), a member of the Fabaceae family, is common throughout tropical and subtropical regions of Asia, Africa, Australia, and America with hot and humid climates, where it is planted as a shade and/or ornamental tree or for binding sand [1, 2]. Pollens from the Fabaceae family have been reported as an important source of pollinosis in the United States, European countries, and Asia [1–3]. Moreover, the inhalation of* A. farnesiana* pollen is one of the main causes of respiratory allergic diseases in semiarid countries such as Iran, Saudi Arabia, and the United Arab Emirates, where the frequency of sensitization ranges from 25% to 48% [2, 4–7].

Howlett et al. reported a high level of cross-reactivity between* Acacia* (wattle) and* Lolium perenne *(rye grass) pollen using the radioallergosorbent test (RAST) [8]. The results showed that IgE molecules which bound to* Acacia* pollen proteins also bound to* L. perenne* pollen extracts. In spite of a high rate of sensitization to* Acacia* pollen in Iran and neighboring countries, to our knowledge, there are few studies about the characterization of* A. farnesiana* pollen extract, and cross-reactivity of this plant with the five most allergenic pollens in arid and semiarid areas (*Prosopis juliflora*,* Salsola kali*,* Amaranthus retroflexus, Chenopodium album,* and* Kochia scoparia*) is not well documented [2, 5, 9, 10].

The recognition of allergenic components of pollens is essential for component-resolved diagnosis, the design of patient-specific immunotherapy, and the explanation of sensitization mechanisms to various allergens [11, 12]. In this study, we evaluate proteins of* Acacia* pollen extract which are specifically reactive to the immunoglobulin E (IgE) of pollen-allergic patients and the IgE cross-reactivity among* A. farnesiana *and the selected plants using* in vivo* and* in vitro* assessments.

## 2. Materials and Methods

### 2.1. Preparation of Extract

Polleniferous materials were collected from* A. farnesiana's* flowers during February–May throughout Ahvaz city, a tropical region in southwest Iran with a tropical climate and a population of more than 1.4 million [2].

Collection and processing of pollen materials was done carefully by trained pollen collectors. Pollen grains were separated by passing the dried materials through different sieves (100, 200, and 300 meshes) successively. The final fine powder was subjected to a purity check for pollen content using a microscope. Pollen materials with more than 95% pollen and less than 5% floral parts of the same plant were taken for antigen extraction.

Pollen materials were defatted using repeated changes of diethyl ether. Pollen was extracted as described previously [10]. In brief, two grams of pollen was mixed with 10 mL phosphate-buffered saline (PBS) 0.01 M (pH 7.4) by continuous stirring for 18 h at 4°C. The extract was centrifuged at 16,000 g, filtered through a 0.22 *μ*m membrane under sterile conditions, and dialyzed against 10 mM phosphate buffer. The extract was then freeze-dried. The protein content of the extract was measured by Bradford's method [13].

### 2.2. Patients and Skin Prick Test

Forty-two respiratory allergic patients were enrolled in this study presented to the Immunology and Allergy Department of Ahvaz Jundishapur University of Medical Sciences. The patients were asked to complete a detailed questionnaire. They were considered as having a history of allergy if they reported at least one eye, nasal, or respiratory symptom to common allergens such as house dust, domestic animals, food, or pollen. The patients were also evaluated by a clinical examination and a skin prick test (SPT) with common aeroallergens. Ten healthy subjects who presented negative SPTs and no specific IgE to the* A. farnesiana *pollen extract were assigned as negative controls. The human ethics committee of the institute approved the study protocol with informed written consent from each patient.

Skin prick tests were performed by an experienced nurse under physician's supervision. In this test,* A. farnesiana *and the most allergenic pollen extracts (*Prosopis juliflora, Salsola kali, Amaranthus retroflexus, Chenopodium album,* and* Kochia scoparia*) were put on the patients' inner forearms and irritation of the epidermis was caused by prick method. The result was observed after 15 minutes. Next, the mean diameter of wheal reaction in every patient was measured and compared with negative (glycerol saline) and positive (histamine, 10 mg/mL) controls. Patients with a wheal diameter >3 mm were considered positive compared to negative and positive controls and were asked to donate a serum sample. Serum samples of patients were stored at −20°C before use.

### 2.3. Enzyme-Linked Immunosorbent Assays (ELISAs)

Total serum IgE levels were measured by means of a commercially available ELISA kit according to the manufacturer's instructions (Radim, Italy).

To measure the levels of specific IgE to* A. farnesiana *pollen in patients' sera, an indirect ELISA was developed as described earlier [14]. Briefly, 0.2 *μ*g of* A. farnesiana *pollen extract in 100 *μ*L carbonate buffer (15 mM Na_2_CO_3_ and 35 mM NaHCO_3_, pH 9.6) was incubated at 4°C overnight per well of a 96-well microtiter plate (Nunc MaxiSorp, Denmark). Each well was then blocked for 1 h at 37°C with 150 *μ*L of 2% BSA in PBS followed by incubation for 3 h with 100 *μ*L of serum at room temperature with shaking. Each well was then incubated for 2 h at room temperature with 1 : 1000 dilution of biotinylated goat anti-human IgE antibody (Nordic Immunology Co., Netherlands) in 1% BSA. Each incubation step was followed by five washes with PBS-T (PBS containing 0.05% Tween 20). Wells were added by 100 *μ*L of a 1 : 5000 dilution of horseradish peroxidase-conjugated streptavidin (Bio-Rad, USA). Following five washes, 100 *μ*L of chromogenic substrate was added to each well and the plate was incubated for 15 min in the dark. The plate was read at 450 nm with an ELISA reader. Optical density (OD_450_) greater than four times the median values of the negative controls was considered to be positive.

### 2.4. Total Extract Preparation

Pollens from* P. juliflora* (mesquite),* S. kali*,* A. retroflexus*,* C. album*, and* K. scoparia* were prepared from polleniferous materials and then their extracts were prepared as described above [10]. The protein content of each extract was then determined using Bradford's method [13].

### 2.5. SDS-PAGE and IgE-Immunoblotting

Sodium dodecyl sulfate polyacrylamide gel electrophoresis (SDS-PAGE) of* Acacia* pollen extract (60 *μ*g) was performed according to Laemmli [15] using 12.5% or 15% acrylamide separation gels under reducing and nonreducing conditions. Reducing and nonreducing sample buffers were the same except that the final reducing sample buffer contained 5% (vol/vol) 2-mercaptoethanol (2-ME). Separated protein bands from the electrophoresis of* A. farnesiana* pollen extract were electrotransferred to polyvinylidene difluoride (PVDF) membranes (Immobilon P, Millipore Corp., Bedford, MA, US), as described earlier [10]. In brief, after washing and blocking, membranes were incubated with a 1/5 dilution of serum pool or individual sera from patients with* A. farnesiana* allergy or with control sera (1 : 5 dilutions). Biotinylated anti-human IgE (Nordic Immunology Co., Netherlands) (1 : 500 v/v in 1% BSA) was added to the blotted membrane strips and incubated for 2 h at room temperature. The unbound antibodies were removed from blots by washing with PBS and followed by incubation with 1 : 10000 v/v in BSA1% HRP-linked streptavidin (Sigma-Aldrich, USA) for 2 h at room temperature. The bound enzymatic activity of horseradish peroxidase was detected by high-sensitivity liquid diaminobenzidine (Liquid DAB^+^) chromogen (DAKO, Denmark).

### 2.6. ELISA Inhibition

ELISA inhibition assays were performed as described above, except that a pooled serum (1 : 2 v/v) from* A. farnesiana* allergic patients (numbers 2, 7, 21, 24, and 36) was preincubated for one hour at room temperature with either 1000, 100, 10, 1, 0.1, or 0.01 *μ*g of the selected pollen extract (including* P. juliflora, S. kali, A. retroflexus*,* C. album,* and* K. scoparia*) as inhibitors or with BSA as a negative control. Percentage of inhibition was calculated using the following formula: (OD of sample without inhibitor − OD of sample with inhibitor/OD of sample without inhibitor) × 100.

### 2.7. IgE-Immunoblotting Inhibition

To study the cross-reactivity between* A. farnesiana *pollen and selected allergenic plants, the IgE-immunoblot inhibition experiment was performed. Reducing SDS-PAGE resolved* A. farnesiana* pollen proteins were transferred to PVDF membrane. After blocking, membrane strips were kept for 3 h at room temperature with a mix of 100 *μ*L of pooled sera (1 : 5 v/v) (from patients 2, 7, 21, 24, and 36) which were preincubated with pollen extracts from* P. juliflora, S. kali, A. retroflexus*,* C. album,* and* K. scoparia*, as well as BSA (as negative control).

## 3. Results

### 3.1. Patients

Forty-two patients, 20 males and 22 females (mean age, 33.60 ± 14.47 years; age range 10–70 years), were included in the study (Table 1). All patients suffered from respiratory allergies and seasonal rhinitis without asthma. The patients were all positive by skin prick test with* A. farnesiana*,* P. juliflora, S. kali, A. retroflexus*,* C. album,* and* K. scoparia* extracts (Table 1). A serum pool of 5 nonallergic subjects was used as a negative control.

### 3.2. Skin Prick Test

Mean diameters of positive wheal sizes were* A. farnesiana*: 6.69 ± 1.17 mm,* P. juliflora*: 7.57 ± 1.50 mm,* S. kali*: 9.95 ± 2.81 mm,* A. retroflexus*: 9.74 ± 4.79 mm,* C. album*: 10.19 ± 3.79 mm, and* K. scoparia*: 8.9 ± 3.70 mm. Positive correlation coefficients were attained between the* A. farnesiana* and* P. juliflora *(*r* = 0.64, *P* < 0.01),* A. farnesiana* and* S. kali* (*r* = 0.07, *P* = 0.6),* A. farnesiana* and* A. retroflexus *(*r* = 0.1, *P* = 0.4)*, A. farnesiana* and* C. album* (*r* = 0.06, *P* = 0.6), and* A. farnesiana* and* K. scoparia* (*r* = 0.007, *P* = 0.9) pollen extracts for wheal diameter on the SPT (ANOVA procedure).

### 3.3. Protein and Allergenic Profile

**Figure 1:**
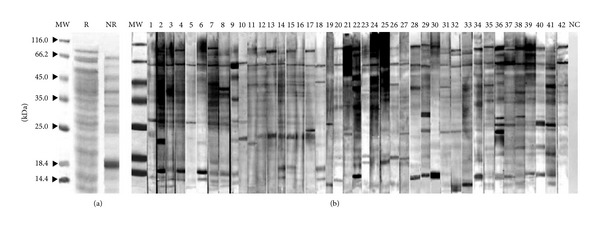
(a) Coomassie Brilliant Blue stained SDS-PAGE of the crude extract of* A. farnesiana* pollen in reducing and nonreducing conditions on 12.5% acrylamide gel. Lane MW, molecular weight marker (Fermentas, Lithuania); R, reducing condition; NR, nonreducing condition. (b) Immunoblotting of* Acacia* pollen extract (with reducing SDS-PAGE). Each strip was first blotted with* Acacia *pollen extract. All strips were then incubated with allergic patients' sera and detected for IgE reactive protein bands. Lane MW, low molecular weight. NC, negative control.

The protein composition of* A. farnesiana* extract was analyzed by Coomassie Brilliant Blue staining (Figure 1(a)). The reducing SDS-PAGE separation of the pollen extract showed several resolved protein bands in the* A. farnesiana* extract with molecular weights in the range of approximately 12 to 85 kDa. The most prominent bands had MWs of approximately 50, 66, and 85 kDa. Other predominant bands were identified at 12, 15, 20, 23, 28, 37, and 45 kDa. Nonreducing SDS-PAGE of* Acacia* pollen extract showed eight prominent protein bands with estimated molecular weights between 15 and 66 kDa (Figure 1(a)).

IgE reactivity of the separated protein bands from the electrophoresis of* Acacia* pollen extract was determined via immunoblotting assays. Specific IgE binding fractions probed with sera from all forty-two allergic patients are shown in Figure 1(b). The results showed several IgE reactive bands ranging from about 12 to 85 kDa. Figure 1(b) shows the apparent MW of each protein fraction and the prevalence of each one among all forty-two allergic patients. The most frequent IgE reactive bands among the patients' sera were approximately 66 and 85 kDa. However, there were other IgE reactive protein bands among patients' sera with molecular weights 15, 20, 23, 28, 39, 45, and 50 kDa (Figure 1(b)). No band was detected when a negative control serum pool was assayed.

### 3.4. Specific IgE Levels and Inhibition ELISA

**Figure 2:**
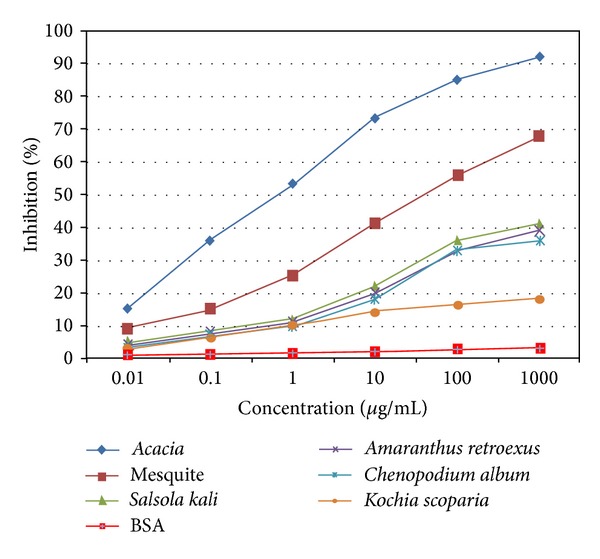
Inhibition of IgE binding to* Acacia* pollen extract by ELISA using pollen extract from the most allergenic plants, mesquite,* S. kali, A. retroflexus*,* C. album,* and* K. scoparia*. Control experiments were performed with BSA.

The sera of forty-two allergic patients were evaluated for specific IgE binding to total* Acacia* pollen extract. All allergic patients had detectable specific IgE levels to the total extract of* Acacia* pollen (Table 1). These results are consistent with those obtained in the IgE-immunoblotting assays using the pollen extract of* Acacia* (Figure 1(b)). To investigate cross-reactivity and allergenic potency among* Acacia* and* P. juliflora, S. kali, A. retroflexus, C. album, *and* K. scoparia*, ELISA inhibition with their extracts was done (Figure 2). Almost complete inhibition was attained with 100 *μ*g/mL of* Acacia* pollen extract as positive control. Preincubation of pooled serum with high concentration (1000 *μ*g/mL) of* P. juliflora* revealed significant inhibition of IgE binding to allergenic proteins of* A. farnesiana* pollen extract (68%) (Figure 2).

### 3.5. Immunoblot Inhibition Assays

**Figure 3:**
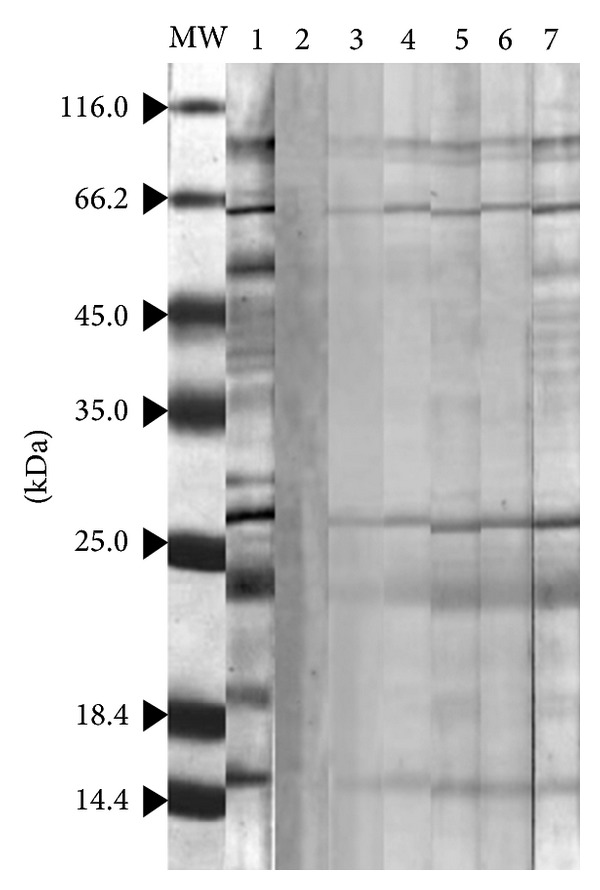
Immunoblotting inhibition assays. Lane MW, molecular weight marker (Fermentas, Lithuania). Lane 1,* Acacia* protein strip incubated with pooled serum without inhibitor (negative control). Lane 2,* Acacia* protein strip incubated with pooled serum containing 70 *μ*g of* Acacia* pollen extract as inhibitor (positive control). Lane 3,* Acacia* protein strip incubated with pooled serum containing 70 *μ*g* P. juliflora* as inhibitor. Lane 4,* Acacia* protein strip incubated with pooled serum containing 70 *μ*g* S. kali* as inhibitor. Lane 5,* Acacia* protein strip incubated with pooled serum containing 70 *μ*g* A. retroflexus* as inhibitor. Lane 6,* Acacia* protein strip incubated with pooled serum containing 70 *μ*g* C. album* as inhibitor. Lane 7,* Acacia* protein strip incubated with pooled serum containing 70 *μ*g* K. scoparia* as inhibitor.

In order to evaluate the IgE cross-reactivity between* Acacia *and five other allergenic pollen extracts, an immunoblot inhibition was carried out with the* Acacia *pollen extract as the solid phase. As shown in Figure 3, IgE reactivity to most of the allergenic proteins of* Acacia *pollen was inhibited when the* Acacia *pollen extract was used as an inhibitor (positive control Lane 2). Complete inhibition of IgE binding to 15, 23, 45, and 50 kDa components of* Acacia* extract occurred when the sera were preincubated with* P. juliflora* extract at 70 *μ*g/mL. Meanwhile, the three bands at about 28, 66, and 85 kDa were partially inhibited. However, when* S. kali, A. retroflexus, C. album, *and* K. scoparia* pollen extracts were used as inhibitors, the 20, 39, 45, and 50 kDa bands were completely inhibited, whereas the 15, 23, 28, 66, and 85 kDa were not inhibited (Figure 3).

## 4. Discussion

*Acacia* is one of the major trees throughout arid and semiarid areas of Iran and neighboring countries along the Persian Gulf and the Sea of Oman [2, 16]. SDS-PAGE revealed several bands from the* Acacia *pollen extract with estimated MWs from 12 kDa to 85 kDa (Figure 1(a)). Among those bands, six IgE binding protein fractions with apparent MWs of 85, 66, 39, 45, 28, 23, and 15 kDa were detected from the blot (Figure 1(b)). Moreover, the results of SDS-PAGE showed relative similarity patterns of migration by protein components of the five selected pollens, mainly those of 15–85 kDa (data not shown). In previous studies the proteins with apparent MWs 39, 45, 66, and 85 kDa have been the most allergenic proteins in the selected allergenic weeds in this study [10, 17–19].

In the current study, the immunoblot analysis with individual patient's serum demonstrated diverse IgE reactivity with seven proteins of 15, 23, 28, 39, 45, 66, and 85 kDa as major IgE binding components. However, immunoblots showed that in the allergenic profile of* Acacia *pollen extract the proteins with apparent MWs of 66 and 85 kDa are the major reactive proteins.

The results of SDS-PAGE revealed that the overall pattern of migration of* Acacia* pollen proteins changed under both reducing conditions. It was thus suggested that probable cysteine residues of the pollen proteins may be associated with interchain disulfide bonds. In previous studies, it was demonstrated that some proteins in pollen extract such as methionine synthase of* S. kali* or* A. retroflexus* were partially degraded into two fragments with approximate MWs of 45 and 39 kDa [19, 20]. Taken together, these observations suggested that some proteins in* Acacia* pollen extract are susceptible to degradation as a result of proteolysis or exposure to a reducing condition. Moreover, it is possible that the number or size of the products of these proteins' degradation depends on the conditions of pollen extract preparation and storage. Nevertheless, additional studies are required to elucidate the patterns of degradation and the number and the size of cleavage products.

Cross-reactivity among* Acacia* pollen components with* Lolium perenne* pollen allergens has been described [8].

Several studies reported that proteins with apparent MWs of 45 and 66 kDa are allergenic in the pollen extracts of mesquite [18] and the selected member of the Chenopodiaceae family [10, 19]. The results of immunoblotting inhibition revealed that the IgE binding reactivity of the allergenic proteins with 12, 20, 39, and 45 from the* A. farnesiana *pollen extract was partially inhibited by all four pollen extracts and also partially with* K. scoparia *pollen extract. As shown in Figure 3, IgE reactivity to most of the allergenic proteins of* A. farnesiana *pollen was inhibited when the* P. juliflora *pollen extract was used as an inhibitor. This indicates a significant IgE cross-reactivity between the two pollens and is also in line with the results of the SPTs (see below).

The results of SPTs indicated highly significant correlations between the wheal diameters from the* A. farnesiana *pollen extract and those from* P. juliflora* pollen extracts (*r* = 0.64, *P* < 0.01). These results suggest an extensive IgE cross-reactivity among these pollens and are concurrent with the results of immunoblotting and inhibition experiments. These results collectively suggest that in these pollen extracts, protein components with MWs range 45 to 66 kDa may play a greater role in cross-reactivity compared to others. Earlier studies had also indicated one of the most allergenic proteins of* S. kali* pollen, named Sal k 1, with a MW of 40–43 kDa [21, 22]. Sal k 1 displayed pectin methylesterase (PME) properties and is considered a major allergen of* S. kali* pollen [14, 21, 22].

In general, the knowledge of pollen cross-reactivity is crucial for diagnostics as well as formulation of immunotherapy vaccines. Cross-reactivity among pollens belonging to the same genus and/or different genera has been demonstrated earlier [23]. Some cross-reactive proteins have been genetically engineered and are found to have potential for use in immunotherapy [24].

Another dominant IgE binding protein band with an estimated MW of 15 kDa was also detected by immunoblotting of* Acacia* pollen extract (Figure 1(b)). Earlier, the allergens belonging to the profilin family with apparent MWs of 14 to 15 kDa were found from* S. kali *(Sal k 4),* A. retroflexus* (Ama r 2), and* C. album* (Che a 2) pollens [25–27]. It may be that the 15 kDa- protein of* Acacia* pollen is homologous with the 15-kDa IgE reactive band in these plants. However, further studies are required to prove the nature of this allergenic protein of* Acacia* pollen.

## 5. Conclusion

In conclusion,* A. farnesiana* pollen is a potent allergen source with several IgE binding components. The observations altogether suggest a close allergenic relationship between* Acacia* and mesquite. Regarding the extensive cross-reactivity between these two trees and the abundance of them in tropical and subtropical regions, identification and production of the recombinant forms of common allergens of these pollens may lead to the exploration of new guidelines for diagnostic, therapeutic, and preventive purposes. Efforts are now underway to clone cDNAs encoding allergenic cross-reactive proteins from* Acacia* pollen.

## Figures and Tables

**Table 1:** Clinical characteristics, total and specific IgE values, and skin reactivity of allergic patients.

Sample number	Age (years)/sex^1^	Symptoms^2^	*Acacia *specific IgE^3^ (OD)	Total IgE (IU/mL)	Diameters (mm) of the papules obtained by prick test^4^					
					*A. farnesiana *	*P. juliflora *	*S. kali *	*A. retroflexus *	*C. album *	*K. scoparia *
Patients										
1	34/M	L, N, E	0.9	181	7	5	8	9	10	9
2	41/M	N, E	2.33	453	8	9	10	30	11	11
3	39/F	L, N	2.71	156	5	7	16	11	10	9
4	14/F	L, N, E	1.11	165	7	9	7	5	14	11
5	12/M	L, N, E	1.10	123	5	6	4	3	2	0
6	10/M	L, N, E	1.19	313	6	5	10	10	9	8
7	29/F	L, N, E	1.89	345	7	8	8	7	11	5
8	43/F	N, E	1.98	234	6	7	10	12	11	12
9	21/M	N, E	2.07	381	7	9	10	10	12	11
10	26/F	N, E	0.89	225	5	7	10	9	10	11
11	26/F	L, N, E	1.02	468	5	5	10	7	12	8
12	23/F	L, N, E	0.85	265	6	8	13	9	10	10
13	30/M	N, E	1.63	167	7	7	10	7	8	9
14	21/F	L, N, E	1.50	275	7	9	10	10	11	12
15	70/M	L, N, E	1.97	187	6	6	12	11	15	10
16	43/F	N, E	1.85	212	8	7	12	8	11	10
17	32/F	L, N, E	1.94	250	6	8	10	11	13	15
18	46/F	L, N, E	1.52	261	5	7	10	7	9	10
19	34/F	N, E	1.42	301	5	6	5	5	4	0
20	13/M	N, E	2.01	463	8	9	12	5	8	6
21	22/M	N	2.21	280	9	8	8	8	6	7
22	17/F	L, N, E	2.02	302	8	10	8	7	5	5
23	61/M	N	1.04	186	5	6	9	10	9	10
24	66/M	L, N, E	2.10	293	10	12	9	8	6	7
25	10/M	L, N	1.83	220	8	7	4	4	5	0
26	33/M	L, N	1.55	214	7	7	10	10	9	9
27	40/F	L, N	1.65	122	5	5	10	9	11	9
28	53/F	N	1.87	200	7	9	8	8	9	10
29	46/M	N, E	1.91	192	7	6	10	9	10	10
30	43/M	L, N, E	2.15	284	7	7	8	7	9	5
31	19/F	L, N, E	1.92	187	8	7	9	8	9	6
32	36/F	L, N, E	1.89	156	7	8	15	23	25	15
33	19/F	L, N, E	1.29	360	7	10	10	9	10	10
34	37/F	N, E	1.93	267	7	8	12	12	11	11
35	32/M	N, E	1.87	228	7	9	8	9	10	10
36	51/M	N, E	2.11	358	7	8	14	13	11	12
37	28/F	L, N, E	1.60	294	6	8	12	11	11	10
38	29/M	L, N, E	1.96	304	6	7	18	16	18	14
39	44/M	N, E	2.02	418	6	8	10	8	9	9
40	40/M	N, E	2.12	315	8	9	13	18	16	17
41	38/F	N	2.09	480	7	8	8	9	9	6
42	40/F	N, E	1.72	257	6	7	8	7	9	5
Controls										
44	28/F	—	0.09	32	**0**	**0**	**0**	**0**	**0**	**0**
45	45/F	—	0.05	12	**0**	**0**	**0**	**0**	**0**	**0**
46	32/M	—	0.06	88	**0**	**0**	**0**	**0**	**0**	**0**
47	38/M	—	0.10	65	**0**	**0**	**0**	**0**	**0**	**0**
48	22/M	—	0.05	22	**0**	**0**	**0**	**0**	**0**	**0**

^1^M: male; F: female.

^2^L: lungs symptoms (breathlessness, tight chest, cough, wheeze); N: nose symptoms (sneezing, runny, blocked); E: eyes symptoms (itching, dry).

^3^Levels of specific IgE to *A. retroflexus* pollen extract by ELISA (optical density at 450 nm).

^4^The mean wheal diameters are displayed in mm. Histamine diphosphate (10 mg/mL) positive control; glycerin-negative control.
